# Psychological impact of caregiving: anxiety and depression levels among caregivers of patients with pressure ulcers

**DOI:** 10.1590/1980-220X-REEUSP-2024-0380en

**Published:** 2025-11-17

**Authors:** Nur Simsek Yurt, Erdinc Yavuz

**Affiliations:** 1 Samsun Training and Research Hospital, Clinic of Family Medicine, Samsun, Turkey.; 2 Samsun University Faculty of Medicine, Department of Family Medicine, Samsun, Turkey.

**Keywords:** Pressure Ulcer, Caregivers, Anxiety, Depression, Home Care Services

## Abstract

**Objective:**

This study aimed to assess the anxiety and depression levels among caregivers of patients with pressure ulcers and explored associated factors.

**Materials and Methods:**

This cross-sectional study was conducted between July and August 2024, in Samsun, Turkey. The study population consisted of caregivers of patients with pressure ulcers registered with the Home Care Services of Samsun Training and Research Hospital. Patient data were obtained from medical records, while caregiver characteristics and psychological status were assessed through face-to-face interviews using standardized anxiety and depression scales. Statistical analyses were performed, with p < 0.05 considered significant.

**Results:**

The study included 122 patient-caregiver pairs. Patients had a mean age of 76.35 ± 13.50 years, with 66.4% female, while caregivers averaged 53.56 ± 11.82 years, with 82.8% female. Caregivers’ anxiety and depression scores were 12.0 ± 10.6 and 12.6 ± 10.1, respectively. Higher anxiety (p = 0.039) and depression (p = 0.002) were observed in caregivers of patients with advanced-stage pressure ulcers. Greater psychological distress was also noted in caregivers providing more than 12 hours of daily care, delivering continuous care, lacking personal income, or having chronic illness (p < 0.05).

**Conclusion:**

Caregivers of patients with severe pressure ulcers experience significant anxiety and depression, highlighting the need for targeted psychological support. Interventions should prioritize caregivers facing prolonged caregiving demands, chronic illness, or financial hardship to protect their mental well-being.

## INTRODUCTION

A pressure ulcer is a wound that develops in the skin and underlying tissue due to prolonged pressure and/or friction, typically over a bony prominence^(1)^. The skin covering bony areas of the body, including the soles of the feet, ankles, hips, and tailbone, is frequently affected by these ulcers. Persistent pressure, along with friction and shear, are contributing factors in the development of pressure ulcers^(2)^. When the epidermis and underlying tissues are compressed between an external surface and bone for extended periods, continuous pressure impairs blood circulation. This leads to tissue damage, which can range from mild (Stage I) to severe (Stage IV), involving muscle and bone^(3)^. Individuals with restricted mobility due to medical conditions, such as paralysis or prolonged bed rest, are at the highest risk^(4)^. The prevalence of pressure ulcers has been reported at 12.3–18% in hospitals and 27% in elderly care facilities^(5,6,7)^. Preventative measures include regular skin assessments, adequate nutrition, and repositioning to alleviate pressure. While minor ulcers can be managed with wound care and dressings, severe cases may require surgical intervention^(8)^.

Pressure ulcers have a profound impact on both patients and their caregivers, affecting their physical, psychological, and social well-being. These wounds can lead to infections, chronic pain, prolonged hospital stays, and rehabilitation challenges^(3)^. Patients with severe ulcers often require long-term care, which places considerable demands on caregivers. Caregivers play a crucial role in wound management, hygiene maintenance, and ensuring patient mobility, making them essential in pressure ulcer care^(4)^. The physical strain of repositioning patients, concerns about causing pain during dressing changes, and witnessing a loved one’s decline can exacerbate stress and emotional distress^(9)^. Additionally, caregivers may struggle with social isolation and financial burdens due to the time-intensive nature of care^(10)^.

Caregivers can be broadly categorized into family caregivers and professional caregivers. Family caregivers, most commonly spouses or children and less frequently distant relatives, provide unpaid care at home and often face emotional and financial burdens. In cases where family support is unavailable or insufficient, caregiving responsibilities may be assumed by paid caregivers, who frequently encounter significant emotional and physical challenges associated with pressure ulcer management. Studies indicate that both groups experience anxiety, depression, and burnout, with family caregivers reporting higher levels of emotional exhaustion^(11,12)^. In addition, caregivers of individuals with chronic conditions have been shown to experience higher rates of anxiety (20–40%) and depression (15–35%) compared to the general population^(13,14,15)^. However, the unique challenges posed by pressure ulcer management, including prolonged wound care and its emotional toll, require further investigation.

Comprehensive care for pressure ulcer patients involves a multidisciplinary approach, including physicians, nurses, dietitians, and physiotherapists^(16)^. Home care services play a critical role in supporting both patients and caregivers. These services provide medical, psychological, and social support, helping caregivers navigate the demands of long-term care^(17)^. Professional healthcare teams can alleviate caregiver burden by offering education on effective wound care techniques, emotional support, and strategies to improve overall well-being^(18)^. Addressing both the physical and psychological aspects of caregiving is essential to enhance the quality of life for both caregivers and patients.

This study aimed to assess the anxiety and depression levels of caregivers of patients with pressure ulcers and to examine the factors associated with these symptoms.

## MATERIALS AND METHODS

### Study Design and Participants

This study is a cross-sectional, descriptive, and analytical study conducted between July 1, 2024, and August 15, 2024, in Samsun, a metropolitan city located in the Black Sea region of Turkey. The study was carried out with caregivers of patients with pressure ulcers registered at the Home Care Services unit of Samsun Training and Research Hospital, a major healthcare facility providing extensive home care services in the region. The inclusion criteria were being the primary caregiver of a patient with a pressure ulcer receiving home healthcare services and agreeing to participate in the study. Caregivers with incomplete data regarding home healthcare services, those diagnosed with mood disorders and undergoing medical treatment as determined by a specialist physician, and those who declined to participate were excluded from the research.

### Sample Size and Data Collection

A total of 168 patients with pressure ulcers were regularly visited by home healthcare services. The minimum required sample size was calculated as 118 participants based on a 95% confidence interval (α = 0.05), an estimated prevalence of 50% (which provides the maximum sample size), and a 5% margin of error (d = 0.05).

Demographic and clinical data of the patients, such as age, gender, chronic diseases, bedbound status, duration of bed confinement, stage of pressure ulcer (stage I–IV), ulcer location (sacral, ischial, heels, elbows, shoulders-back), duration of the pressure ulcer (in months), and method of feeding (oral, nasogastric (NG), percutaneous endoscopic gastrostomy (PEG)), were recorded on a data form created by reviewing patient files.

For the caregivers, primary data were collected on their age, gender, relationship status (spouse, child, other relatives, paid caregiver), daily caregiving duration, total caregiving duration, caregiving method (periodic/continuous), chronic disease status, and anxiety and depression symptoms, which were evaluated through face-to-face interviews using the Beck Anxiety Inventory (BAI) and the Beck Depression Inventory (BDI).

### Data Collection Tools

The Beck Depression Inventory (BDI) was developed by Beck et al. in 1961^(19)^ to assess depression levels in participants, and its Turkish validity and reliability study was conducted by Hisli in 1989^(20)^. It consists of 21 items, each with four options on a Likert-type scale. Based on the score, the degree of depression is categorized as normal (0–9 points), mild depression (10–16 points), moderate depression (17–29 points), and severe depression (30–63 points).

The Beck Anxiety Inventory (BAI) is a self-report scale that determines the frequency of anxiety symptoms^(21)^. The Turkish validity and reliability study was conducted by Ulusoy et al.^(22)^, and the Cronbach alpha value was reported as 0.93. It consists of 21 items, scored on a Likert-type scale ranging from 0 to 3. Higher scores on the scale indicate greater severity of anxiety experienced by the individual. Scores ranging from 0–7 are considered normal, 8–15 indicate mild anxiety, 16–25 indicate moderate anxiety, and 26–63 indicate severe anxiety.

In Turkey, the BDI and BAI are not limited to use by psychologists; they can also be administered by other healthcare professionals, such as psychiatrists, nurses, and social workers, who have received training in psychological assessments. Various studies conducted in Turkey have demonstrated that these scales are effectively utilized by different healthcare providers to evaluate the mental health of caregivers in diverse settings^(23,24)^.

### Ethics Statement

Ethics committee approval was obtained with the Ethics Committee Decision No. GOKAEK/2024/12/3 of Samsun University Non-Interventional Clinical Research Ethics Committee. This study was conducted in accordance with the principles outlined in the Declaration of Helsinki. Informed consent was obtained from all participants prior to data collection. The study adhered to the STROBE (Strengthening the Reporting of Observational Studies in Epidemiology) guidelines to ensure comprehensive and transparent reporting of methods and results.

### Statistical Analysis

Data were analyzed using SPSS 26.0 (Statistical Package for the Social Sciences) software. The Kolmogorov-Smirnov and Shapiro-Wilk tests were applied to determine the normality distribution of the data. For comparing the non-normally distributed BAI and BDI scores, as well as two-category variables, the Mann-Whitney U test was used. For comparing variables with more than two categories, the Kruskal-Wallis test was employed. Descriptive statistics, including mean ± standard deviation, minimum-maximum values, frequency distribution, and percentages, were presented. Statistical significance was accepted at p < 0.05.

## RESULTS

The study included 122 patients with pressure ulcers and 122 primary caregivers registered in home care services. Among the patients, 66.4% (n = 81) were female, with an average age of 76.35 ± 13.50 years (range: 31–101 years). The most common comorbidities included hypertension (35.2%, n = 43), cardiovascular disease (32.0%, n = 39), dementia (32.0%, n = 39), chronic kidney failure (17.2%, n = 21), and cerebrovascular disease (15.6%, n = 19). In terms of feeding methods, 73.0% (n = 89) were orally fed, 23.8% (n = 29) utilized percutaneous endoscopic gastrostomy (PEG), and 3.3% (n = 4) were fed via nasogastric tube (NG). Over half of the patients (54.1%, n = 66) had been bedridden for less than a year. Regarding the stage of pressure ulcers, 6.6% (n = 8) had stage I, 27.9% (n = 34) had stage II, 45.9% (n = 56) had stage III, and 19.7% (n = 24) had stage IV. The ulcer sites were most frequently located in the sacral region (52.5%, n = 64), followed by the heels (13.1%, n = 16), ischial region (13.1%, n = 16), shoulder/back area (10.7%, n = 13), and other regions (9.0%, n = 11) (Table 1).

**Table 1:** Demographic and clinical characteristics of patients with pressure ulcers – Samsun, Turkey, 2024.

Variables	n	%
Gender		
Female	81	66.4
Male	41	33.6
Chronic diseases*		
Hypertension	43	35.2
Diabetes	39	32.0
Renal failure	21	17.2
Cardiovascular disease	39	32.0
Chronic pulmonary disease	12	9.8
Cerebrovascular disease	19	15.6
Dementia	39	32.0
Oncological disease	9	7.4
Other**	21	17.2
Duration of bedridden		
< 1 year	66	54.1
1-5 years	39	32.0
>5 years	17	13.9
Nutrition method		
Oral	89	73.0
NG	4	3.3
PEG	29	23.8
Location of pressure ulcer		
Sacral	64	52.5
Heel	16	13.1
Ischial	16	13.1
Shoulder-Back	13	10.7
Elbow	2	1.6
Other	11	9.0
Stage of pressure ulcer		
Stage I	8	6.6
Stage II	34	27.9
Stage III	56	45.9
Stage IV	24	19.7
Duration of pressure ulcer		
0–3 months	60	49.2
4–12 months	41	33.6
13–24 months	21	17.2
Patient's income status		
Yes	96	78.7
No	26	21.3

*Participants may have multiple chronic diseases, so the total exceeds the number of participants.

**Thyroid disease, epilepsy, Parkinson's disease, benign prostate disease, chronic rheumatic diseases, liver disease, musculoskeletal diseases.

Abbreviations: NG, Nasogastric tube; PEG, Percutaneous endoscopic gastrostomy.

Among caregivers, 82.8% (n = 101) were female, with a mean age of 53.56 ± 11.82 years (range: 23–78 years), and the majority (65.6%, n = 80) were aged between 45–64 years. Notably, 41.0% (n = 50) of caregivers were the patient’s children, while 36.9% (n = 45) were other relatives. Nearly all caregivers (88.5%, n = 108) provided care for more than 12 hours daily, and continuous care was reported by the same proportion. Approximately half (44.3%, n = 54) of caregivers had their own income, while 54.9% (n = 67) reported having a chronic disease. Caregivers’ mean scores for the Beck Anxiety Inventory (BAI) and Beck Depression Inventory (BDI) were 12.04 ± 10.6 and 12.64 ± 10.05, respectively, with 54.9% and 56.6% exhibiting anxiety and depressive symptoms, respectively (Table 2).

**Table 2:** Demographic and clinical characteristics of caregivers – Samsun, Turkey, 2024.

Variables	n	%
Age Group		
18–44	23	18.8
45–64	80	65.6
≥ 65	19	15.6
Gender		
Female	101	82.8
Male	21	17.2
Relationship		
Spouse	19	15.6
Child	50	41.0
Other relative	45	36.8
Paid caregiver	8	6.6
Daily care duration		
1–12 hours	14	11.5
13–24 hours	108	88.5
Income status		
Yes	54	44.3
None	68	55.7
Type of care		
Periodic	14	11.5
Continuous	108	88.5
Chronic disease		
Yes	67	54.9
None	55	45.1
BAI category		
Normal	55	45.1
Mild	28	22.9
Moderate	25	20.5
Severe	14	11.5
BDI category		
Normal	53	43.4
Mild	39	32.0
Moderate	17	13.9
Severe	13	10.7

Abbreviations: BAI. Beck Anxiety Inventory; BDI. Beck Depression Inventory.

Comparative analysis revealed that caregivers of patients with stage IV pressure ulcers had significantly higher BAI and BDI scores (p = 0.039, p = 0.002, respectively). Conversely, caregivers of patients fed via PEG or NG tubes reported significantly lower BAI scores (p = 0.041) (Table 3).

**Table 3:** Relationship between anxiety and depression levels of caregivers and clinical characteristics of patients – Samsun, Turkey, 2024.

Variables	BAI	p	BDI	p
Nutrition method				
Oral	13.28 ± 10.79	0.041^a^	13.56 ± 10.27	0.133^a^
NG	8.50 ± 7.55		8.50 ± 7.77	
PEG	8.72 ± 10.20		10.38 ± 9.42	
Location of pressure ulcer				
Sacral	11.83 ± 10.74	0.464^a^	11.17 ± 9.35	0.211^a^
Heel	13.31 ± 10.55		13.87 ± 9.32	
Ischial	11.81 ± 11.28		14.81 ± 12.60	
Shoulder-Back	12.54 ± 11.09		13.85 ± 11.50	
Elbow	1.00 ± 0.00		5.00 ± 4.24	
Other	13.18 ± 11.06		16.18 ± 9.66	
Stage of pressure ulcer				
Stage 1	10.63 ± 7.13	0.039^a^	7.13 ± 6.29	0.002^a^
Stage 2	9.79 ± 8.37		9.65 ± 7.32	
Stage 3	10.50 ± 9.52		12.07 ± 9.51	
Stage 4	19.29 ± 14.14		20.04 ± 12.06	
Duration of pressure ulcer				
0–3 months	11.13 ± 10.67	0.299^a^	11.93 ± 9.30	0.525^a^
4–12 months	12.34 ± 11.26		12.51 ± 10.42	
13–24 months	14.05 ± 9.76		14.90 ± 11.52	
Duration of bedridden				
< 1 year	11.59 ± 11.01	0.626^a^	12.35 ± 10.26	0.731^a^
1-5 years	13.44 ± 11.60		13.69 ± 10.86	
>5 years	10.59 ± 6.66		11.35 ± 7.25	
Patient income				
Yes	12.51 ± 11.08	0.499^b^	12.67 ± 10.18	0.856^b^
None	10.31 ± 9.10		12.54 ± 9.80	

^a^Kruskal Wallis Test

^b^Mann-Whitney U Test

Abbreviations: BAI, Beck Anxiety Inventory; BDI, Beck Depression Inventory.

Caregiver-specific characteristics also influenced BAI and BDI scores: caregivers who provided care for more than 12 hours daily, offered continuous care, lacked personal income, or had a chronic disease demonstrated significantly higher BAI scores (p = 0.002, p = 0.001, p = 0.002, p < 0.001, respectively). Paid caregivers had notably lower BAI scores (p = 0.005). Similar trends were observed in BDI scores, with caregivers meeting the same criteria (more than 12 hours of care, continuous care, no personal income, chronic disease) exhibiting significantly higher BDI scores (p = 0.030, p = 0.005, p = 0.017, p < 0.001, respectively) (Table 4).

**Table 4:** Relationship between anxiety and depression levels of caregivers and caregiver characteristics – Samsun, Turkey, 2024.

Variables	BAI	p	BDI	p
Age groups				
18–44	10.61 ± 8.94	0.642^a^	11.09 ± 8.77	0.732^a^
45–64	13.08 ± 11.77		13.28 ± 11.11	
65≤	9.42 ± 6.94		11.84 ± 6.23	
Gender				
Female	12.97 ± 11.22	0.096^b^	13.29 ± 10.38	0.090^b^
Male	7.57 ± 6.04		9.52 ± 7.85	
Relationship				
Spouse	11.47 ± 7.11	0.005^a^	12.00 ± 4.15	0.110^a^
Child	13.30 ± 11.98		13.64 ± 11.24	
Other relative	12.67 ± 10.60		13.04 ± 10.85	
Paid caregiver	2.00 ± 2.14		5.63 ± 3.85	
Daily care duration				
1–12	4.64 ± 4.36	0.002^b^	7.71 ± 7.53	0.030^b^
13–24	13.00 ± 10.90		13.28 ± 10.20	
Income status				
Yes	8.56 ± 8.11	0.002^b^	9.69 ± 6.82	0.017^b^
No	14.81 ± 11.70		14.99 ± 11.55	
Care type				
Periodic	4.43 ± 4.11	0.001^b^	6.14 ± 4.69	0.005^b^
Continuous	13.03 ± 10.89		13.48 ± 10.27	
Chronic disease				
Yes	15.91 ± 11.75	< 0.001^b^	16.03 ± 10.77	< 0.001^b^
No	7.33 ± 6.78		8.51 ± 7.31	

^a^Kruskal Wallis Test

^b^Mann-Whitney U Test

Abbreviations: BAI, Beck Anxiety Inventory; BDI, Beck Depression Inventory.

## DISCUSSION

This study, we comprehensively analyzed the levels of anxiety and depression among caregivers of patients with pressure ulcers receiving home care services, examining their associations with various demographic and clinical characteristics of both patients and caregivers. The findings contribute to existing research by detailing the psychological health profile of caregivers for patients with pressure ulcers.

Pressure ulcers in home care patients were most frequently observed in the sacral region and were predominantly stage III in severity. This finding aligns with data commonly reported in the literature^(3,25)^. The higher prevalence of advanced-stage pressure ulcers may be linked to insufficient pressure ulcer management and prolonged immobility. Additionally, patients regularly monitored for wound care at our center tend to present with more advanced-stage ulcers. Closer monitoring of pressure ulcer risk factors and implementing early intervention strategies are crucial in preventing progression to more severe ulcer stages^(3)^. Nutritional challenges faced by these patients can negatively impact the wound healing process. As a result, the use of alternative feeding methods, such as tube feeding, in this patient group may be associated with an increased need for both wound management and overall care requirements^(26)^. In this study, while the majority of patients were orally fed, 23.8% were fed via PEG, highlighting the advanced healthcare needs of this patient group. Lower anxiety levels observed among caregivers of patients fed via NG and PEG tubes suggest reduced emotional burnout, potentially due to the systematic and predictable nature of care required for these patients.

In this study, caregivers of patients with stage IV pressure ulcers exhibited higher levels of both anxiety and depression. Depressive symptoms among caregivers increased in correlation with the progression of pressure ulcer stages. These findings align with Fox’s study^(27)^, which highlights the relationship between the severity of pressure ulcers and increased psychological distress, emphasizing the impact of physical illness severity on mental well-being. Similarly, Qian et al.^(4)^ demonstrated that caregiver stress increases in direct relation to the progression of pressure ulcer stages. These findings indicate that caregivers of patients with advanced-stage pressure ulcers experience higher levels of psychological distress, likely related to the increased challenges associated with the patients’ growing care needs. Psychological support interventions for caregivers, particularly those caring for patients with advanced-stage pressure ulcers, are therefore essential.

The majority of caregivers in this study were female. This gender disparity in caregiving roles reflects the gender- based distribution of caregiving responsibilities and aligns with findings from previous studies^(28,29)^. In a study by Alhammadi et al.^(30)^, where caregivers of patients with pressure ulcers participated in an educational program, 91% of the caregivers were female. Although female caregivers exhibited higher levels of anxiety and depression symptoms compared to male caregivers in this study, the differences were not statistically significant. Similarly, studies in the literature that have reported no significant gender differences in the psychological well-being of caregivers, aligning with these results^(29,31)^. Despite female bearing a greater emotional and physical burden in the caregiving process, gender-based differences in psychological effects are not always evident. This observation could be attributed to individual variations in coping mechanisms or differences in adaptation to caregiving roles, influenced by personal and environmental factors.

Caregivers providing long hours of daily care, offering continuous care, lacking personal income, and suffering from chronic disease were found to have significantly higher levels of anxiety and depressive symptoms. Lee and Lee^(32)^ have also noted a potential correlation between the prolonged and challenging process of caregiving for individuals with pressure ulcers and increased levels of stress and depression among caregivers. Qian et al.^(4)^ reported that caregivers of patients with pressure ulcers who had chronic diseases and longer caregiving durations experienced increased levels of fatigue and a decrease in their life satisfaction. These findings suggest that the psychological health of caregivers is significantly influenced by factors such as the duration of caregiving, economic status, and chronic disease. Caregivers who experience prolonged caregiving periods, economic hardship, and chronic health conditions appear at heightened risk for increased stress, fatigue, and depression. These results underscore the need for supportive programs and interventions aimed at mitigating these risks for caregivers facing challenging care situations.

### Limitations

The cross-sectional design of this study limits the ability to establish causal relationships, as the findings reflect only a specific time point. Additionally, being a single-center study, the generalizability of the results is constrained, and certain clinical and psychosocial factors may have been overlooked in the data collection process. The use of self-report scales to assess anxiety and depression among caregivers also introduces the potential for bias.

## CONCLUSION

In conclusion, patients with pressure ulcers and their caregivers represent a high-risk group that requires specialized attention from healthcare systems. As the stage of pressure ulcers progresses, the psychological burden on caregivers increases. The impact of caregivers’ socioeconomic status and chronic disease on their anxiety and depression levels should be considered when planning targeted interventions for these individuals. Regular psychological counseling and preventive interventions for caregivers are strongly recommended. Our research highlights the need for more comprehensive strategies to improve pressure ulcer management and caregiver support within the framework of home care services.

## Data Availability

The datasets generated and/or analyzed during the current study are available from the corresponding author on reasonable request.
