# Supplementary material for: Psychological impact of caregiving: anxiety and depression levels among caregivers of patients with pressure ulcers
Source: Rev Esc Enferm USP. 2025 Nov 17;59:e20240380. doi: 10.1590/1980-220X-REEUSP-2024-0380en (PMC12700353; doi:10.1590/1980-220X-REEUSP-2024-0380en)
Supplement: Translation

# Translation: Impacto psicológico do cuidado nos níveis de ansiedade e depressão entre cuidadores de pacientes com úlceras por pressão

**DOI:** 10.1590/1980-220X-REEUSP-2024-0380pt

**Authors:** Nur Simsek Yurt, Erdinc Yavuz

**Affiliations:** 1 Samsun Training and Research Hospital, Clinic of Family Medicine, Samsun, Turkey.; 2 Samsun University Faculty of Medicine, Department of Family Medicine, Samsun, Turkey.

**Keywords:** Úlcera por pressão, Cuidadores, Ansiedade, Depressão, Serviços de Assistência Domiciliar

## Abstract

**Objetivo:**

Este estudo teve como objetivo avaliar os níveis de ansiedade e depressão entre cuidadores de pacientes com úlceras por pressão e investigar os fatores associados.

**Materiais e Métodos:**

Trata-se de um estudo transversal realizado entre julho e agosto de 2024 em Samsun, Turquia. A população do estudo foi composta por cuidadores de pacientes com úlceras por pressão registrados nos Serviços de Assistência Domiciliar do Hospital de Ensino e Pesquisa de Samsun. Os dados dos pacientes foram obtidos de prontuários médicos, enquanto as características e o estado psicológico dos cuidadores foram avaliados por meio de entrevistas presenciais utilizando escalas padronizadas de ansiedade e depressão. As análises estatísticas foram realizadas considerando significância para p < 0,05.

**Resultados:**

O estudo incluiu 122 pares de pacientes e cuidadores. A média de idade dos pacientes foi de 76,35 ± 13,50 anos, sendo 66,4% do sexo feminino, enquanto os cuidadores tinham média de 53,56 ± 11,82 anos, com 82,8% do sexo feminino. As pontuações médias de ansiedade e depressão dos cuidadores foram de 12,0 ± 10,6 e 12,6 ± 10,1, respectivamente. Foram observados níveis mais elevados de ansiedade (p = 0,039) e depressão (p = 0,002) entre cuidadores de pacientes com úlceras em estágio avançado. Maior sofrimento psicológico também foi identificado em cuidadores que prestavam mais de 12 horas de cuidado diário, ofereciam cuidados contínuos, não possuíam renda própria ou apresentavam doenças crônicas (p < 0,05).

**Conclusão:**

Cuidadores de pacientes com úlceras por pressão graves apresentam níveis significativos de ansiedade e depressão, ressaltando a necessidade de suporte psicológico direcionado. As intervenções devem priorizar cuidadores submetidos a demandas prolongadas de cuidado, com doenças crônicas ou dificuldades financeiras, visando proteger sua saúde mental.

## INTRODUÇÃO

Úlcera por pressão é uma ferida que se desenvolve na pele e no tecido subjacente devido à pressão prolongada ou fricção, geralmente sobre uma proeminência óssea^(1)^. A pele que cobre as áreas ósseas do corpo, incluindo solas dos pés, tornozelos, quadris e cóccix, é frequentemente afetada por essas úlceras. A pressão persistente, juntamente com o atrito e o cisalhamento, contribuem para o desenvolvimento de úlceras por pressão^(2)^.

Quando a epiderme e os tecidos subjacentes são comprimidos entre uma superfície externa e o osso por longos períodos, a pressão contínua prejudica a circulação sanguínea. Isso leva a danos nos tecidos, que podem variar de leves (estágio I) a graves (estágio IV), envolvendo músculos e ossos^(3)^. Indivíduos com mobilidade restrita devido a condições de saúde, como paralisia ou repouso prolongado na cama, correm maior risco^(4)^.

A prevalência de úlceras por pressão foi relatada em 12,3 a 18% em hospitais e 27% em instituições de cuidados a idosos^(5–7)^. As medidas preventivas incluem avaliações regulares da pele, nutrição adequada e reposicionamento para aliviar a pressão. Embora úlceras menores possam ser tratadas com cuidados com feridas e curativos, casos graves podem exigir intervenção cirúrgica^(8)^.

As úlceras por pressão têm impacto profundo tanto nos pacientes quanto em seus cuidadores, afetando seu bem-estar físico, psicológico e social. Essas feridas podem levar a infecções, dor crônica, internações prolongadas e desafios de reabilitação^(3)^. Pacientes com úlceras graves geralmente requerem cuidados de longo prazo, o que exige muito dos cuidadores. Os cuidadores desempenham um papel crucial no tratamento de feridas, manutenção da higiene e garantia da mobilidade do paciente, o que os torna essenciais no tratamento de úlceras por pressão^(4)^. O esforço físico de reposicionar os pacientes, as preocupações em causar dor durante as trocas de curativos e testemunhar o declínio de um ente querido podem exacerbar o estresse e o sofrimento emocional^(9)^. Além disso, os cuidadores podem lutar com o isolamento social e os encargos financeiros devido à natureza prolongada dos cuidados^(10)^.

Os cuidadores podem ser amplamente categorizados em familiares e profissionais. Os cuidadores familiares, mais comumente cônjuges ou filhos e, menos frequentemente, parentes distantes, prestam cuidados não remunerados em casa e muitas vezes enfrentam encargos emocionais e financeiros. Nos casos em que o apoio familiar não está disponível ou é insuficiente, as responsabilidades de cuidar podem ser assumidas por cuidadores remunerados, que também encontram desafios emocionais e físicos significativos associados ao tratamento de úlceras por pressão. Estudos indicam que ambos os grupos experimentam ansiedade, depressão e esgotamento, com os cuidadores familiares relatando níveis mais altos de exaustão emocional^(11,12)^. Além disso, cuidadores de indivíduos com condições crônicas apresentaram taxas mais altas de ansiedade (20–40%) e depressão (15–35%) em comparação com a população em geral^(13–15)^. No entanto, os desafios únicos impostos pelo tratamento da úlcera por pressão, incluindo o tratamento prolongado da ferida e seu impacto emocional, requerem investigação mais aprofundada.

O cuidado integral aos pacientes com úlcera por pressão envolve uma abordagem multidisciplinar que inclui médicos, enfermeiros, nutricionistas e fisioterapeutas^(16)^. Os serviços de atendimento domiciliário desempenham papel crítico no apoio a pacientes e cuidadores. Esses serviços fornecem apoio médico, psicológico e social, ajudando os cuidadores a lidar com as demandas de cuidados de longo prazo^(17)^. As equipes profissionais de saúde podem aliviar a sobrecarga do cuidador, oferecendo educação sobre técnicas eficazes de tratamento de feridas, apoio emocional e estratégias para melhorar seu bem-estar geral^(18)^. Abordar os aspectos físicos e psicológicos do cuidado é essencial para melhorar a qualidade de vida de cuidadores e pacientes.

Este estudo teve como objetivo avaliar os níveis de ansiedade e depressão de cuidadores de pacientes com úlcera por pressão e examinar os fatores associados a esses sintomas.

## MATERIAIS E MÉTODOS

### Desenho do Estudo e Participantes

Estudo transversal, descritivo e analítico realizado entre 1º de julho e 15 de agosto de 2024, em Samsun, uma cidade metropolitana localizada na região do Mar Negro, na Turquia. O estudo foi realizado com cuidadores de pacientes com úlceras por pressão registrados na unidade de Serviços de Assistência Domiciliária do Hospital de Treinamento e Pesquisa de Samsun, uma importante unidade de saúde que oferece serviços de assistência em domicílio extensivos na região.

Os critérios de inclusão foram ser o cuidador principal de um paciente com úlcera por pressão que recebia serviços de saúde domiciliária e concordar em participar do estudo. Foram excluídos da pesquisa os cuidadores com dados incompletos sobre os serviços de saúde domiciliária, aqueles com diagnóstico de transtornos de humor e em tratamento médico determinado por especialista e aqueles que se recusaram a participar.

### Tamanho da Amostra e Coleta de Dados

Um total de 168 pacientes com úlcera por pressão foram visitados regularmente pelos serviços de saúde domiciliária. O tamanho mínimo necessário da amostra foi calculado como 118 participantes com base em um intervalo de confiança de 95% (α = 0,05), prevalência estimada de 50% (que fornece o tamanho máximo da amostra) e margem de erro de 5% (d = 0,05).

Os dados demográficos e clínicos dos pacientes, como idade, sexo, doenças crônicas, estado de acamado, tempo de confinamento no leito, estágio da úlcera por pressão (estágio I a IV), localização da úlcera (sacral, isquiática, calcanhares, cotovelos, ombros-dorso), duração da úlcera por pressão (em meses) e modo de alimentação (oral, nasogástrica, gastrostomia endoscópica percutânea) foram registrados em um formulário criado mediante revisão dos prontuários dos pacientes.

Para os cuidadores, foram coletados dados primários sobre idade, sexo, situação de relacionamento (cônjuge, filho, outros parentes), remunerado ou não, duração diária do cuidado, duração total do cuidado, método de cuidado (periódico/contínuo), estado de doença crônica e sintomas de ansiedade e depressão, que foram avaliados por meio de entrevistas face a face usando o Inventário de Ansiedade de Beck (IAB) e o Inventário de Depressão de Beck (IDB).

### Ferramentas de Coleta de Dados

O IDB foi desenvolvido por Beck et al. em 1961^(19)^ para avaliar os níveis de depressão nos participantes e seu estudo de validade e confiabilidade turco foi conduzido por Hisli em 1989^(20)^. É composto por 21 itens, cada um com quatro opções em uma escala do tipo Likert. Com base na pontuação, o grau de depressão é categorizado em normal (0–9 pontos), depressão leve (10–16 pontos), moderada (17–29 pontos) e grave (30–63 pontos).

O IAB é uma escala de autorrelato que determina a frequência dos sintomas de ansiedade^(21)^. O estudo de validade e confiabilidade turco foi conduzido por Ulusoy et al.^(22)^ e o valor do alfa de Cronbach relatado foi 0,93. É composto por 21 itens, pontuados em uma escala do tipo Likert que varia de 0 a 3. Pontuações mais altas na escala indicam maior gravidade da ansiedade experimentada pelo indivíduo. Pontuações entre 0 a 7 são consideradas normais, 8 a 15 indicam ansiedade leve, 16 a 25, ansiedade moderada e 26 a 63, ansiedade grave.

Na Turquia, o IDB e o IAB não são utilizados somente por psicólogos; também podem ser administrados por outros profissionais de saúde, como psiquiatras, enfermeiros e assistentes sociais que receberam treinamento em avaliações psicológicas. Vários estudos realizados na Turquia demonstraram que essas escalas são efetivamente utilizadas por diferentes profissionais de saúde para avaliar a saúde mental dos cuidadores em diversos ambientes^(23,24)^.

### Aspectos éticos

A aprovação para a realização deste estudo foi obtida mediante decisão nº. GOKAEK/2024/12/3 do Comitê de Ética em Pesquisa Clínica Não Intervencionista da Universidade de Samsun. Foi conduzido de acordo com os princípios descritos na Declaração de Helsinque. O consentimento informado foi obtido de todos os participantes antes da coleta de dados. O estudo aderiu às diretrizes STROBE (*Strengthening the Reporting of Observational Studies in Epidemiology*) para garantir relatórios abrangentes e transparentes de métodos e resultados.

### Análise Estatística

Os dados foram analisados por meio do *software* SPSS 26.0 (*Statistical Package for the Social Sciences*). Os testes de Kolmogorov-Smirnov e Shapiro-Wilk foram aplicados para determinar a distribuição normal dos dados. Para comparar os escores do IAB e do IDB não normalmente distribuídos, bem como as variáveis de duas categorias, foi utilizado o teste U de Mann-Whitney. Para comparação de variáveis com mais de duas categorias, foi empregado o teste de Kruskal-Wallis. Foram ainda apresentadas estatísticas descritivas, incluindo média e desvio padrão, valores mínimo-máximo, distribuição de frequência e porcentagens. A significância estatística foi aceita em p < 0,05.

## RESULTADOS

Participaram do estudo 122 pacientes com úlcera por pressão e 122 cuidadores primários cadastrados em serviços de atenção domiciliária. Entre os pacientes, 66,4% (n = 81) eram do sexo feminino, com média de idade de 76,35 ± 13,50 anos (variação de 31 a 101 anos). As comorbidades mais comuns incluíram hipertensão (35,2%, n = 43), doença cardiovascular (32,0%, n = 39), demência (32,0%, n = 39), insuficiência renal crônica (17,2%, n = 21) e doença cerebrovascular (15,6%, n = 19). Em relação aos métodos de alimentação, 73,0% (n = 89) eram alimentados por via oral, 23,8% (n = 29) utilizavam gastrostomia endoscópica percutânea e 3,3% (n = 4), sonda nasogástrica. Mais da metade dos pacientes (54,1%, n = 66) estava acamada há menos de um ano. Em relação ao estágio das úlceras por pressão, 6,6% (n = 8) apresentaram estágio I, 27,9% (n = 34) estágio II, 45,9% (n = 56) estágio III e 19,7% (n = 24) estágio IV. Os locais mais frequentes das úlceras foram a região sacral (52,5%, n = 64), os calcanhares (13,1%, n = 16), a região isquiática (13,1%, n = 16), a área dos ombros/costas (10,7%, n = 13) e outras regiões (9,0%, n = 11) (Tabela 1).

**Tabela 1:** Características demográficas e clínicas de pacientes com úlcera por pressão – Samsun, Turquia, 2024.

Variáveis	n	%
Gênero		
Feminino	81	66.4
Masculino	41	33.6
Doenças crônicas*		
Hipertensão	43	35.2
Diabetes	39	32.0
Falência renal	21	17.2
Doença cardiovascular	39	32.0
Doença pulmonar crônica	12	9.8
Doença cerebrovascular	19	15.6
Demência	39	32.0
Doença oncológica	9	7.4
Outras**	21	17.2
Tempo de acamado		
< 1 ano	66	54.1
1–5 anos	39	32.0
>5 anos	17	13.9
Método de nutrição		
Oral	89	73.0
Sonda nasogástrica	4	3.3
Gastrostomia endoscópica percutânea	29	23.8
Localização da úlcera por pressão		
Sacral	64	52.5
Calcanhar	16	13.1
Isquiática	16	13.1
Ombro-Costas	13	10.7
Cotovelo	2	1.6
Outra	11	9.0
Estágio da úlcera por pressão		
Estágio I	8	6.6
Estágio II	34	27.9
Estágio III	56	45.9
Estágio IV	24	19.7
Duração da úlcera por pressão		
0–3 meses	60	49.2
4–12 meses	41	33.6
13–24 meses	21	17.2
Renda do paciente		
Sim	96	78.7
Não	26	21.3

*Os participantes podiam ter várias doenças crônicas, portanto, o total excede o número de participantes.

**Doença da tireoide, epilepsia, doença de Parkinson, doença benigna da próstata, doença hepática, doenças reumáticas crônicas, doenças musculoesqueléticas.

Abreviaturas: NG, sonda nasogástrica; PEG, gastrostomia endoscópica percutânea.

Entre os cuidadores, 82,8% (n = 101) era do sexo feminino, com média de idade de 53,56 ± 11,82 anos (variação de 23 a78 anos) e a maioria (65,6%, n = 80) tinha entre 45 e 64 anos. Destaca-se que 41,0% (n = 50) dos cuidadores eram filhos do paciente, enquanto 36,9% (n = 45) eram outros parentes. Quase todos (88,5%, n = 108) prestavam cuidados por mais de 12 horas diárias e o cuidado contínuo foi relatado na mesma proporção. Aproximadamente metade (44,3%, n = 54) possuía renda própria e 54,9% (n = 67) relataram ter doença crônica. Os escores médios dos cuidadores para o IAB e o IDB foram de 12,04 ± 10,6 e 12,64 ± 10,05, respectivamente, com 54,9% e 56,6% apresentando ansiedade e sintomas depressivos, respectivamente (Tabela 2).

**Tabela 2:** Características demográficas e clínicas dos cuidadores – Samsun, Turquia, 2024.

Variáveis	n	%
Faixa etária		
18–44	23	18.8
45–64	80	65.6
≥ 65	19	15.6
Gênero		
Feminino	101	82.8
Masculino	21	17.2
Relação		
Esposo	19	15.6
Filho	50	41.0
Outro parente	45	36.8
Cuidador remunerado	8	6.6
Duração diária do cuidado		
1–12 horas	14	11.5
13–24 horas	108	88.5
Renda		
Sim	54	44.3
Não	68	55.7
Tipo de atendimento		
Periódico	14	11.5
Contínuo	108	88.5
Doença crônica		
Sim	67	54.9
Não	55	45.1
Categoria IAB		
Normal	55	45.1
Leve	28	22.9
Moderada	25	20.5
Grave	14	11.5
Categoria IDB		
Normal	53	43.4
Leve	39	32.0
Moderada	17	13.9
Grave	13	10.7

Abreviaturas: IAB. Inventário de Ansiedade de Beck; IDB. Inventário de Depressão de Beck.

A análise comparativa revelou que os cuidadores de pacientes com úlcera por pressão estágio IV apresentaram escores IAB e IDB significativamente maiores (p = 0,039, p = 0,002, respectivamente). Por outro lado, os cuidadores de pacientes alimentados por gastrostomia endoscópica percutânea ou sonda nasogástrica relataram escores IAB significativamente mais baixos (p = 0,041) (Tabela 3).

**Tabela 3:** Relação entre os níveis de ansiedade e depressão dos cuidadores e as características clínicas dos pacientes – Samsun, Turquia, 2024.

Variáveis	IAB	p	IDB	p
Método de nutrição				
Oral	13.28 ± 10.79	0.041^a^	13.56 ± 10.27	0.133^a^
NG	8.50 ± 7.55		8.50 ± 7.77	
Gastrostomia endoscópica percutânea	8.72 ± 10.20		10.38 ± 9.42	
Localização da úlcera por pressão				
Sacral	11.83 ± 10.74	0.464^a^	11.17 ± 9.35	0.211^a^
Calcanhar	13.31 ± 10.55		13.87 ± 9.32	
Isquiático	11.81 ± 11.28		14.81 ± 12.60	
Ombro-Costas	12.54 ± 11.09		13.85 ± 11.50	
Cotovelo	1.00 ± 0.00		5.00 ± 4.24	
Outro	13.18 ± 11.06		16.18 ± 9.66	
Estágio da úlcera por pressão				
Estágio 1	10.63 ± 7.13	0.039^a^	7.13 ± 6.29	0.002^a^
Estágio 2	9.79 ± 8.37		9.65 ± 7.32	
Estágio 3	10.50 ± 9.52		12.07 ± 9.51	
Estágio 4	19.29 ± 14.14		20.04 ± 12.06	
Duração da úlcera por pressão				
0–3 meses	11.13 ± 10.67	0.299^a^	11.93 ± 9.30	0.525^a^
4–12 meses	12.34 ± 11.26		12.51 ± 10.42	
13–24 meses	14.05 ± 9.76		14.90 ± 11.52	
Duração de acamado				
< 1 ano	11.59 ± 11.01	0.626^a^	12.35 ± 10.26	0.731^a^
1–5 anos	13.44 ± 11.60		13.69 ± 10.86	
>5 anos	10.59 ± 6.66		11.35 ± 7.25	
Renda				
Sim	12.51 ± 11.08	0.499^b^	12.67 ± 10.18	0.856^b^
Nenhum	10.31 ± 9.10		12.54 ± 9.80	

^a^Teste de Kruskal-Wallis

^b^Teste U de Mann-Whitney

Abreviaturas: IAB, Inventário de Ansiedade de Beck e IDB, Inventário de Depressão de Beck.

As características específicas do cuidador também influenciaram os escores do IAB e do IDB. Cuidadores que prestaram cuidados por mais de 12 horas diárias, ofereceram cuidados contínuos, não tinham renda pessoal ou tinham uma doença crônica demonstraram escores IAB significativamente mais altos (p = 0,002, p = 0,001, p = 0,002, p < 0,001, respectivamente). Os cuidadores remunerados apresentaram escores IAB notavelmente mais baixos (p = 0,005). Tendências semelhantes foram observadas nos escores do IDB, com os cuidadores que atendiam os mesmos critérios (mais de 12 horas de cuidado, cuidados contínuos, sem renda pessoal e com doença crônica) exibindo escores significativamente mais altos (p = 0,030, p = 0,005, p = 0,017, p < 0,001, respectivamente) (Tabela 4).

**Tabela 4:** Relação entre os níveis de ansiedade e depressão dos cuidadores e características dos cuidadores – Samsun, Turquia, 2024.

Variáveis	IAB	p	IDB	p
Faixas etárias				
18–44	10.61 ± 8.94	0.642^a^	11.09 ± 8.77	0.732^a^
45–64	13.08 ± 11.77		13.28 ± 11.11	
65≤	9.42 ± 6.94		11.84 ± 6.23	
Gênero				
Feminino	12.97 ± 11.22	0.096^b^	13.29 ± 10.38	0.090^b^
Masculino	7.57 ± 6.04		9.52 ± 7.85	
Relação				
Esposo	11.47 ± 7.11	0.005^a^	12.00 ± 4.15	0.110^a^
Filho	13.30 ± 11.98		13.64 ± 11.24	
Outro parente	12.67 ± 10.60		13.04 ± 10.85	
Cuidador remunerado	2.00 ± 2.14		5.63 ± 3.85	
Duração diária do cuidado				
1–12	4.64 ± 4.36	0.002^b^	7.71 ± 7.53	0.030^b^
13–24	13.00 ± 10.90		13.28 ± 10.20	
Renda				
Sim	8.56 ± 8.11	0.002^b^	9.69 ± 6.82	0.017^b^
Não	14.81 ± 11.70		14.99 ± 11.55	
Tipo de cuidado				
Periódico	4.43 ± 4.11	0.001^b^	6.14 ± 4.69	0.005^b^
Contínuo	13.03 ± 10.89		13.48 ± 10.27	
Doença crônica				
Sim	15.91 ± 11.75	< 0.001^b^	16.03 ± 10.77	< 0.001^b^
Não	7.33 ± 6.78		8.51 ± 7.31	

^a^Teste de Kruskal-Wallis

^b^Teste U de Mann-Whitney

Abreviaturas: IAB, Inventário de Ansiedade de Beck e IDB, Inventário de Depressão de Beck.

## DISCUSSÃO

Neste estudo, foram analisados de forma abrangente os níveis de ansiedade e depressão entre cuidadores de pacientes com úlcera por pressão atendidos em serviços de atenção domiciliária, examinando suas associações com várias características demográficas e clínicas de pacientes e cuidadores. Os resultados contribuem para a pesquisa existente, detalhando o perfil de saúde psicológica dos cuidadores de pacientes com úlceras por pressão.

As úlceras por pressão em pacientes em cuidados domiciliários foram mais frequentemente observadas na região sacral e foram predominantemente de estágio III de gravidade. Esse achado alinha-se com os dados comumente relatados na literatura^(3,25)^.

A maior prevalência de úlceras por pressão em estágio avançado pode estar ligada ao manejo insuficiente da úlcera por pressão e à imobilidade prolongada. Além disso, os pacientes monitorados regularmente para tratamento de feridas no serviço de saúde estudado tendem a apresentar úlceras em estágio mais avançado. O monitoramento mais próximo dos fatores de risco para úlceras por pressão e a implementação de estratégias de intervenção precoce são cruciais para prevenir a progressão para estágios mais graves da úlcera^(3)^.

Os desafios nutricionais enfrentados por esses pacientes podem impactar negativamente o processo de cicatrização de feridas. Como resultado, o uso de métodos alternativos de alimentação nesse grupo de pacientes, como a alimentação por sonda, pode estar associado a maior necessidade de tratamento de feridas e requisitos gerais de cuidados^(26)^. Neste estudo, enquanto a maioria dos pacientes foi alimentada por via oral, 23,8% foram alimentados via gastrostomia endoscópica percutânea, destacando as necessidades avançadas de saúde desse grupo de pacientes. Níveis mais baixos de ansiedade observados entre os cuidadores de pacientes alimentados por gastrostomia endoscópica percutânea e sonda nasogástrica sugerem esgotamento emocional reduzido, potencialmente devido à natureza sistemática e previsível dos cuidados necessários para esses pacientes.

Neste estudo, os cuidadores de pacientes com úlcera por pressão estágio IV apresentaram níveis mais elevados de ansiedade e depressão. Os sintomas depressivos entre os cuidadores aumentaram em correlação direta com a progressão dos estágios da úlcera por pressão. Esses achado corroboram os de Fox^(27)^, que destaca a relação entre a gravidade das úlceras por pressão e o aumento do sofrimento psicológico, enfatizando o impacto da gravidade da doença física no bem-estar mental. Qian et al.^(4)^ também demonstraram que o estresse do cuidador aumenta em relação direta com a progressão dos estágios da úlcera por pressão. Esses achados indicam que os cuidadores de pacientes com úlceras por pressão em estágio avançado experimentam níveis mais altos de sofrimento psicológico, provavelmente relacionados aos desafios associados às crescentes necessidades de cuidados dos pacientes. Intervenções de apoio psicológico para cuidadores, particularmente aqueles que cuidam de pacientes com úlceras por pressão em estágio avançado são, portanto, essenciais.

A maioria dos cuidadores deste estudo era do sexo feminino. Essa disparidade de gênero nas funções de cuidador reflete a distribuição das responsabilidades de cuidado com base no gênero e alinha-se aos achados de estudos anteriores^(28,29)^. Em investigação de Alhammadi et al.^(30)^, dentre os cuidadores de pacientes com úlceras por pressão que participaram de um programa educacional, 91% era do sexo feminino.

Embora os cuidadores do sexo feminino deste estudo tenham apresentado níveis mais elevados de sintomas de ansiedade e depressão em comparação com os do sexo masculino, as diferenças não foram estatisticamente significativas. Da mesma forma, a literatura não relata diferenças significativas de gênero no bem-estar psicológico dos cuidadores^(29,31)^. Apesar de as mulheres suportarem maior carga emocional e física no processo de cuidado, as diferenças de gênero nos efeitos psicológicos nem sempre são evidentes. Essa observação pode ser atribuída a variações individuais nos mecanismos de enfrentamento ou diferenças na adaptação aos papéis de cuidador, influenciadas por fatores pessoais e ambientais.

Verificou-se que os cuidadores que prestam longas horas de cuidados diários, oferecem cuidados contínuos, não têm renda pessoal e sofrem de doenças crônicas apresentam níveis significativamente mais altos de ansiedade e sintomas depressivos. Lee e Lee^(32)^ também observaram correlação potencial entre o processo prolongado e desafiador de cuidar de indivíduos com úlceras por pressão e o aumento dos níveis de estresse e depressão entre os cuidadores. Qian et al.^(4)^ constataram que cuidadores de pacientes com úlceras por pressão que tinham doenças crônicas e maior duração dos cuidados experimentaram níveis aumentados de fadiga e diminuição da satisfação com a vida. Esses achados sugerem que a saúde psicológica dos cuidadores é significativamente influenciada por fatores como duração do cuidado, status econômico e doença crônica. Os cuidadores que passam por períodos prolongados de cuidado, dificuldades econômicas e condições crônicas de saúde parecem ter maior risco de aumento do estresse, fadiga e depressão. Esses resultados ressaltam a necessidade de programas e intervenções de apoio que visem mitigar esses riscos para os cuidadores que enfrentam situações desafiadoras de cuidado.

### Limitações

O desenho transversal deste estudo limita a capacidade de estabelecer relações causais, pois os achados refletem apenas um ponto de tempo específico. Além disso, por ser um estudo unicêntrico, a generalização dos resultados é restrita e certos fatores clínicos e psicossociais podem ter sido negligenciados no processo de coleta de dados. O uso de escalas de autorrelato para avaliar ansiedade e depressão entre cuidadores também introduz o potencial de viés.

## CONCLUSÃO

Pacientes com úlcera por pressão e seus cuidadores representam um grupo de alto risco que requer atenção especializada dos sistemas de saúde. À medida que o estágio das úlceras por pressão progride, a carga psicológica sobre os cuidadores aumenta. O impacto do status socioeconômico e da doença crônica dos cuidadores em seus níveis de ansiedade e depressão deve ser considerado ao planejar intervenções direcionadas para esses indivíduos. Aconselhamento psicológico regular e intervenções preventivas para cuidadores são fortemente recomendados. Esta pesquisa destaca a necessidade de estratégias mais abrangentes para melhorar o gerenciamento de úlceras por pressão e o apoio ao cuidador no âmbito dos serviços de atendimento domiciliário.

## Data Availability

Os conjuntos de dados gerados e/ou analisados durante o presente estudo estão disponíveis com o autor correspondente mediante solicitação razoável.

# Translation: Impacto psicológico del cuidado: niveles de ansiedad y depresión entre los cuidadores de pacientes con úlceras por presión

**DOI:** 10.1590/1980-220X-REEUSP-2024-0380es

**Authors:** Nur Simsek Yurt, Erdinc Yavuz

**Affiliations:** 1 Samsun Training and Research Hospital, Clinic of Family Medicine, Samsun, Turkey; 2 Samsun University Faculty of Medicine, Department of Family Medicine, Samsun, Turkey

**Keywords:** Úlcera por Presión, Cuidadores, Ansiedad, Depresión, Servicios de Atención de Salud a Domicilio

## Abstract

**Objetivo:**

Este estudio tuvo como objetivo evaluar los niveles de ansiedad y depresión entre los cuidadores de pacientes con úlceras por presión y explorar los factores asociados.

**Materiales y Métodos:**

Se realizó un estudio transversal entre julio y agosto de 2024 en Samsun, Turquía. La población del estudio estuvo compuesta por cuidadores de pacientes con úlceras por presión registrados en los Servicios de Atención Domiciliaria del Hospital de Capacitación e Investigación de Samsun. Los datos de los pacientes se obtuvieron de los registros médicos, mientras que las características y el estado psicológico de los cuidadores se evaluaron mediante entrevistas presenciales utilizando escalas estandarizadas de ansiedad y depresión. Se realizaron análisis estadísticos considerando significativo un valor de p < 0,05.

**Resultados:**

El estudio incluyó 122 pares de pacientes y cuidadores. La edad media de los pacientes fue de 76,35 ± 13,50 años, siendo el 66,4% mujeres, mientras que los cuidadores tuvieron una media de 53,56 ± 11,82 años, con un-82,8% de mujeres. Las puntuaciones medias de ansiedad y depresión de los cuidadores fueron 12,0 ± 10,6 y 12,6 ± 10,1, respectivamente. Se observaron niveles más altos de ansiedad (p = 0,039) y depresión (p = 0,002) en cuidadores de pacientes con úlceras por presión en etapas avanzadas. Además, se registró mayor malestar psicológico en cuidadores que proporcionaban más de 12 horas de cuidado diario, brindaban atención continua, carecían de ingresos propios o presentaban enfermedades crónicas (p < 0,05).

**Conclusión:**

Los cuidadores de pacientes con úlceras por presión graves experimentan niveles significativos de ansiedad y depresión, lo que subraya la necesidad de apoyo psicológico específico. Las intervenciones deben priorizar a los cuidadores sometidos a demandas prolongadas de atención, que padecen enfermedades crónicas o dificultades económicas, para proteger su bienestar mental.

## INTRODUCCIÓN

Una úlcera por presión es una herida que se desarrolla en la piel y el tejido subyacente debido a una presión y/o fricción prolongadas, normalmente sobre una prominencia ósea^(1)^. La piel que cubre las zonas óseas del cuerpo, incluidas las plantas de los pies, los tobillos, las caderas y el coxis, se ve afectada con frecuencia por estas úlceras. La presión persistente, junto con la fricción y el cizallamiento, son factores que contribuyen al desarrollo de úlceras por presión^(2)^. Cuando la epidermis y los tejidos subyacentes se comprimen entre una superficie externa y el hueso durante períodos prolongados, la presión continua perjudica la circulación sanguínea. Esto provoca daños en los tejidos, que pueden variar de leves (etapa I) a graves (etapa IV), afectando a los músculos y los huesos^(3)^. Las personas con movilidad restringida debido a afecciones médicas, como parálisis o reposo prolongado en cama, corren el mayor riesgo^(4)^. La prevalencia de las úlceras por presión se ha situado entre el 12,3% y el 18% en los hospitales y el 27% en los centros de atención a personas mayores^(5–7)^. Las medidas preventivas incluyen evaluaciones periódicas de la piel, una nutrición adecuada y cambios de posición para aliviar la presión. Mientras que las úlceras leves pueden tratarse con cuidados de la herida y apósitos, los casos graves pueden requerir intervención quirúrgica^(8)^.

Las úlceras por presión tienen un profundo impacto tanto en los pacientes como en sus cuidadores, ya que afectan a su bienestar físico, psicológico y social. Estas heridas pueden provocar infecciones, dolor crónico, estancias hospitalarias prolongadas y dificultades de rehabilitación^(3)^. Los pacientes con úlceras graves suelen requerir cuidados a largo plazo, lo que supone una carga considerable para los cuidadores. Los cuidadores desempeñan un papel crucial en el tratamiento de las heridas, el mantenimiento de la higiene y la movilidad de los pacientes, lo que los hace esenciales en el cuidado de las úlceras por presión^(4)^. El esfuerzo físico que supone cambiar de posición a los pacientes, la preocupación por causarles dolor durante los cambios de vendajes y ser testigos del deterioro de un ser querido pueden exacerbar el estrés y la angustia emocional^(9)^. Además, los cuidadores pueden sufrir aislamiento social y cargas económicas debido al tiempo que requiere el cuidado^(10)^.

Los cuidadores pueden clasificarse, en términos generales, en cuidadores familiares y cuidadores profesionales. Los cuidadores familiares, que suelen ser cónyuges o hijos y, con menos frecuencia, parientes lejanos, prestan cuidados no remunerados en el hogar y a menudo se enfrentan a cargas emocionales y económicas. En los casos en que no se dispone de apoyo familiar o este es insuficiente, las responsabilidades del cuidado pueden recaer en cuidadores remunerados, que a menudo se enfrentan a importantes retos emocionales y físicos asociados al tratamiento de las úlceras por presión. Los estudios indican que ambos grupos experimentan ansiedad, depresión y agotamiento, y los cuidadores familiares reportan niveles más altos de agotamiento emocional^(11,12)^. Además, se ha demostrado que los cuidadores de personas con enfermedades crónicas experimentan tasas más altas de ansiedad (20–40%) y depresión (15–35%) en comparación con la población general^(13–15)^. Sin embargo, los retos únicos que plantea el tratamiento de las úlceras por presión, incluido el cuidado prolongado de las heridas y su impacto emocional, requieren una investigación más profunda.

La atención integral de los pacientes con úlceras por presión implica un enfoque multidisciplinario, que incluye a médicos, enfermeros, dietistas y fisioterapeutas^(16)^. Los servicios de atención domiciliaria desempeñan un papel fundamental en el apoyo tanto a los pacientes como a los cuidadores. Estos servicios proporcionan apoyo médico, psicológico y social, ayudando a los cuidadores a hacer frente a las exigencias de los cuidados a largo plazo^(17)^. Los equipos de atención médica profesionales pueden aliviar la carga de los cuidadores ofreciéndoles formación sobre técnicas eficaces de cuidado de las heridas, apoyo emocional y estrategias para mejorar el bienestar general^(18)^. Abordar tanto los aspectos físicos como psicológicos del cuidado es esencial para mejorar la calidad de vida tanto de los cuidadores como de los pacientes.

El objetivo de este estudio fue evaluar los niveles de ansiedad y depresión de los cuidadores de pacientes con úlceras por presión y examinar los factores asociados a estos síntomas.

## MATERIALES Y MÉTODOS

### Diseño del Estudio y Participantes

Este estudio es un estudio transversal, descriptivo y analítico realizado entre el 1 de julio de 2024 y el 15 de agosto de 2024 en Samsun, una ciudad metropolitana situada en la región del Mar Negro de Turquía. El estudio se llevó a cabo con cuidadores de pacientes con úlceras por presión registrados en la unidad de Servicios de Atención Domiciliaria del Hospital de Formación e Investigación de Samsun, un importante centro sanitario que presta amplios servicios de atención domiciliaria en la región. Los criterios de inclusión fueron ser el cuidador principal de un paciente con úlcera por presión que recibía servicios de atención médica a domicilio y aceptar participar en el estudio. Se excluyó de la investigación a los cuidadores con datos incompletos sobre los servicios de atención médica a domicilio, a los diagnosticados con trastornos del estado de ánimo y sometidos a tratamiento médico según lo determinado por un médico especialista, y a los que se negaron a participar.

### Tamaño de la Muestra y Recopilación de Datos

Un total de 168 pacientes con úlceras por presión recibían visitas periódicas de los servicios de atención domiciliaria. El tamaño mínimo requerido de la muestra se calculó en 118 participantes, basándose en un intervalo de confianza del 95% (α = 0,05), una prevalencia estimada del 50% (que proporciona el tamaño máximo de la muestra) y un margen de error del 5% (d = 0,05).

Se recopilaron datos demográficos y clínicos de los pacientes, como la edad, el sexo, las enfermedades crónicas, el estado de postración en cama, la duración de la inmovilización en cama, la etapa de la úlcera por presión (etapa I–IV), ubicación de la úlcera (sacra, isquiática, talones, codos, hombros-espalda), duración de la úlcera por presión (en meses) y método de alimentación: oral, nasogástrica (NG), gastrostomía endoscópica percutánea (PEG), se registraron en un formulario de datos creado a partir de la revisión de los expedientes de los pacientes.

En cuanto a los cuidadores, se recolectaron datos primarios sobre su edad, sexo, relación con el paciente (cónyuge, hijo, otros familiares, cuidador remunerado), duración diaria de los cuidados, duración total de los cuidados, método de cuidado (periódico/continuo), estado de enfermedad crónica y síntomas de ansiedad y depresión, que se evaluaron mediante entrevistas presenciales utilizando el Inventario de Ansiedad de Beck (Beck Anxiety Inventory – BAI) y el Inventario de Depresión de Beck (Beck Depression Inventory – BDI).

### Instrumentos de Recolección de Datos

El Inventario de Depresión de Beck (BDI) fue desarrollado por Beck et al. en 1961 para evaluar los niveles de depresión en los participantes^(19)^, y su estudio de validez y fiabilidad en turco fue realizado por Hisli en 1989^(20)^. Consta de 21 ítems, cada uno con cuatro opciones en una escala tipo Likert. Según la puntuación, el grado de depresión se clasifica en normal (0–9 puntos), depresión leve (10–16 puntos), depresión moderada (17–29 puntos) y depresión grave (30–63 puntos).

El Inventario de Ansiedad de Beck (BAI) es una escala de autoinforme que determina la frecuencia de los síntomas de ansiedad^(21)^. El estudio de validez y fiabilidad en turco fue realizado por Ulusoy et al.^(22)^, y el valor alfa de Cronbach fue de 0,93. Consta de 21 ítems, puntuados en una escala tipo Likert que va de 0 a 3. Las puntuaciones más altas en la escala indican una mayor gravedad de la ansiedad experimentada por el individuo. Las puntuaciones que oscilan entre 0 y 7 se consideran normales, las de 8 a 15 indican ansiedad leve, las de 16 a 25 indican ansiedad moderada y las de 26 a 63 indican ansiedad grave.

En Turquía, el BDI y el BAI no se limitan al uso por parte de psicólogos, sino que también pueden ser administrados por otros profesionales de la salud, como psiquiatras, enfermeros y trabajadores sociales, que hayan recibido formación en evaluaciones psicológicas. Diversos estudios realizados en Turquía han demostrado que estas escalas son utilizadas eficazmente por diferentes proveedores de atención médica para evaluar la salud mental de los cuidadores en diversos entornos^(23,24)^.

### Declaración ética

Se obtuvo la aprobación del comité de ética con la Decisión del Comité de Ética n.º GOKAEK/2024/12/3 del Comité de Ética de Investigación Clínica No Intervencionista de la Universidad de Samsun. Este estudio se llevó a cabo de acuerdo con los principios descritos en la Declaración de Helsinki. Se obtuvo el consentimiento informado de todos los participantes antes de la recopilación de datos.

El estudio se ajustó a las directrices STROBE (Strengthening the Reporting of Observational Studies in Epidemiology) para garantizar una presentación completa y transparente de los métodos y los resultados.

### Análisis Estadístico

Los datos se analizaron con el software SPSS 26,0 (Statistical Package for the Social Sciences). Se aplicaron las pruebas de Kolmogorov-Smirnov y Shapiro-Wilk para determinar la distribución normal de los datos. Para comparar las puntuaciones BAI y BDI no distribuidas normalmente, así como las variables de dos categorías, se utilizó la prueba U de Mann-Whitney. Para comparar variables con más de dos categorías, se empleó la prueba de Kruskal-Wallis. Se presentaron estadísticas descriptivas, incluyendo la media ± desviación estándar, los valores mínimos y máximos, la distribución de frecuencias y los porcentajes. Se aceptó la significación estadística con p < 0,05.

## RESULTADOS

El estudio incluyó a 122 pacientes con úlceras por presión y 122 cuidadores principales registrados en los servicios de atención domiciliaria. Entre los pacientes, el 66,4% (n = 81) eran mujeres, con una edad media de 76,35 ± 13,50 años (rango: 31–101 años). Las comorbilidades más comunes fueron hipertensión (35,2%, n = 43), enfermedad cardiovascular (32,0%, n = 39), demencia (32,0%, n = 39), insuficiencia renal crónica (17,2%, n = 21) y enfermedad cerebrovascular (15,6%, n = 19). En cuanto a los métodos de alimentación, el 73,0% (n = 89) se alimentaba por vía oral, el 23,8% (n = 29) utilizaba una gastrostomía endoscópica percutánea (PEG) y el 3,3% (n = 4) se alimentaba mediante una sonda nasogástrica (NG). Más de la mitad de los pacientes (54,1%, n = 66) habían estado postrados en cama durante menos de un año. En cuanto al estadio de las úlceras por presión, el 6,6% (n = 8) presentaba estadio I, el 27,9% (n = 34) presentaba estadio II, el 45,9% (n = 56) presentaba estadio III y el 19,7% (n = 24) presentaba estadio IV. Las úlceras se localizaron con mayor frecuencia en la región sacra (52,5%, n = 64), seguida de los talones (13,1%, n = 16), la región isquiática (13,1%, n = 16), la zona de los hombros/espalda (10,7%, n = 13) y otras regiones (9,0%, n = 11) (Tabla 1).

**Tabla 1:** Características demográficas y clínicas de los pacientes con úlceras por presión – Samsun, Turquia, 2024.

Variables	n	%
Género		
Femenino	81	66,4
Masculino	41	33,6
Enfermedades crónicas*		
Hipertensión	43	35,2
Diabetes	39	32,0
Falla renal	21	17,2
Enfermedad cardiovascular	39	32,0
Enfermedad pulmonar crónica	12	9,8
Enfermedad cerebrovascular	19	15,6
Demencia	39	32,0
Enfermedad oncológica	9	7,4
Otras**	21	17,2
Duración de encamamiento		
< 1 año	66	54,1
1–5 años	39	32,0
>5 años	17	13,9
Método nutricional		
Oral	89	73,0
NG	4	3,3
PEG	29	23,8
Lugar de la úlcera por presión		
Sacra	64	52,5
Talón	16	13,1
Isquiática	16	13,1
Hombro-espalda	13	10,7
Codo	2	1,6
Otro	11	9,0
Estadio de la úlcera por presión		
Estadio I	8	6,6
Estadio II	34	27,9
Estadio III	56	45,9
Estadio IV	24	19,7
Duración de la úlcera por presión		
0–3 meses	60	49,2
4–12 meses	41	33,6
13–24 meses	21	17,2
Estatus de ingresos del paciente		
Tiene	96	78,7
No tiene	26	21,3

*Los participantes pueden padecer varias enfermedades crónicas, por lo que el total supera el número de participantes.

**Enfermedad tiroidea, epilepsia, enfermedad de Parkinson, enfermedad benigna de la próstata, enfermedades reumáticas crónicas, enfermedad hepática, enfermedades musculoesqueléticas.

Siglas: NG, sonda nasogástrica; PEG, gastrostomía endoscópica percutánea.

Entre los cuidadores, el 82,8% (n = 101) eran mujeres, con una edad media de 53,56 ± 11,82 años (rango: 23–78 años), y la mayoría (65,6%, n = 80) tenían entre 45 y 64 años. Cabe destacar que el 41,0% (n = 50) de los cuidadores eran hijos de los pacientes, mientras que el 36,9% (n = 45) eran otros familiares. Casi todos los cuidadores (88,5%, n = 108) prestaban cuidados durante más de 12 horas al día, y la misma proporción informó de que los cuidados eran continuos. Aproximadamente la mitad (44,3%, n = 54) de los cuidadores tenían ingresos propios, mientras que el 54,9% (n = 67) informó de que padecía una enfermedad crónica. Las puntuaciones medias de los cuidadores en el Inventario de Ansiedad de Beck (BAI) y el Inventario de Depresión de Beck (BDI) fueron de 12,04 ± 10,6 y 12,64 ± 10,05, respectivamente, y el 54,9% y el 56,6% mostraron síntomas de ansiedad y depresión, respectivamente (Tabla 2).

**Tabla 2:** Características demográficas y clínicas de los cuidadores – Samsun, Turquia, 2024.

Variables	n	%
Grupos de edad		
18–44	23	18,8
45–64	80	65,6
≥ 65	19	15,6
Género		
Femenino	101	82,8
Masculino	21	17,2
Relación		
Cónyuge	19	15,6
Hijo/a	50	41,0
Otro pariente	45	36,8
Cuidador contratado/a	8	6,6
Duración del cuidado diario		
1–12 horas	14	11,5
13–24 horas	108	88,5
Estatus de ingresos		
Tiene	54	44,3
No tiene	68	55,7
Tipo de cuidado		
Periódico	14	11,5
Continuo	108	88,5
Enfermedad crónica		
Si	67	54,9
No	55	45,1
Categoría de BAI		
Normal	55	45,1
Leve	28	22,9
Moderada	25	20,5
Severa	14	11,5
Categoría de BDI		
Normal	53	43,4
Leve	39	32,0
Moderada	17	13,9
Severa	13	10,7

Siglas: BAI. Inventario de Ansiedad de Beck (Beck Anxiety Inventory); BDI. Inventario de Depresión de Beck (Beck Depression Inventory).

El análisis comparativo reveló que los cuidadores de pacientes con úlceras por presión en estadio IV tenían puntuaciones significativamente más altas en el BAI y el BDI (p = 0,039 y p = 0,002, respectivamente). Por el contrario, los cuidadores de pacientes alimentados mediante sondas PEG o NG reportaron puntuaciones significativamente más bajas en el BAI (p = 0,041) (Tabla 3).

**Tabla 3:** Relación entre los niveles de ansiedad y depresión de los cuidadores y las características clínicas de los pacientes – Samsun, Turquia, 2024.

Variables	BAI	p	BDI	p
Método nutricional				
Oral	13,28 ± 10,79	0,041^a^	13,56 ± 10,27	0,133^a^
NG	8,50 ± 7,55		8,50 ± 7,77	
PEG	8,72 ± 10,20		10,38 ± 9,42	
Lugar de la úlcera por presión				
Sacra	11,83 ± 10,74	0,464^a^	11,17 ± 9,35	0,211^a^
Talón	13,31 ± 10,55		13,87 ± 9,32	
Isquiática	11,81 ± 11,28		14,81 ± 12,60	
Hombro-espalda	12,54 ± 11,09		13,85 ± 11,50	
Codo	1,00 ± 0,00		5,00 ± 4,24	
Otra	13,18 ± 11,06		16,18 ± 9,66	
Estadio de la úlcera por presión				
Estadio 1	10,63 ± 7,13	0,039^a^	7,13 ± 6,29	0,002^a^
Estadio 2	9,79 ± 8,37		9,65 ± 7,32	
Estadio 3	10,50 ± 9,52		12,07 ± 9,51	
Estadio 4	19,29 ± 14,14		20,04 ± 12,06	
Duración de la úlcera por presión				
0–3 meses	11,13 ± 10,67	0,299^a^	11,93 ± 9,30	0,525^a^
4–12 meses	12,34 ± 11,26		12,51 ± 10,42	
13–24 meses	14,05 ± 9,76		14,90 ± 11,52	
Duración de encamamiento				
< 1 año	11,59 ± 11,01	0,626^a^	12,35 ± 10,26	0,731^a^
1–5 años	13,44 ± 11,60		13,69 ± 10,86	
>5 años	10,59 ± 6,66		11,35 ± 7,25	
Ingresos del paciente				
Tiene	12,51 ± 11,08	0,499^b^	12,67 ± 10,18	0,856^b^
No tiene	10,31 ± 9,10		12,54 ± 9,80	

^a^Test Kruskal Wallis

^b^Test Mann-Whitney U.

Siglas: BAI. Inventario de Ansiedad de Beck (Beck Anxiety Inventory); BDI. Inventario de Depresión de Beck (Beck Depression Inventory).

Las características específicas de los cuidadores también influyeron en las puntuaciones del BAI y el BDI: los cuidadores que prestaban cuidados durante más de 12 horas al día, ofrecían cuidados continuos, carecían de ingresos personales o padecían una enfermedad crónica obtuvieron puntuaciones significativamente más altas en el BAI (p = 0,002, p = 0,001, p = 0,002 y p < 0,001, respectivamente). Los cuidadores remunerados obtuvieron puntuaciones notablemente más bajas en la escala BAI (p = 0,005). Se observaron tendencias similares en las puntuaciones del BDI, y los cuidadores que cumplían los mismos criterios (más de 12 horas de cuidados, cuidados continuos, sin ingresos personales, enfermedad crónica) obtuvieron puntuaciones significativamente más altas en el BDI (p = 0,030, p = 0,005, p = 0,017, p < 0,001, respectivamente) (Tabla 4).

**Tabla 4:** Relación entre los niveles de ansiedad y depresión de los cuidadores y las características de los cuidadores – Samsun, Turquia, 2024.

Variables	BAI	p	BDI	p
Grupos de edad				
18–44	10,61 ± 8,94	0,642^a^	11,09 ± 8,77	0,732^a^
45–64	13,08 ± 11,77		13,28 ± 11,11	
65≤	9,42 ± 6,94		11,84 ± 6,23	
Género				
Femenino	12,97 ± 11,22	0,096^b^	13,29 ± 10,38	0,090^b^
Masculino	7,57 ± 6,04		9,52 ± 7,85	
Relación				
Cónyuge	11,47 ± 7,11	0,005^a^	12,00 ± 4,15	0,110^a^
Hijo/a	13,30 ± 11,98		13,64 ± 11,24	
Otro pariente	12,67 ± 10,60		13,04 ± 10,85	
Cuidador contratado/a	2,00 ± 2,14		5,63 ± 3,85	
Duración del cuidado diario				
1–12	4,64 ± 4,36	0,002^b^	7,71 ± 7,53	0,030^b^
13–24	13,00 ± 10,90		13,28 ± 10,20	
Estatus de ingresos				
Tiene	8,56 ± 8,11	0,002^b^	9,69 ± 6,82	0,017^b^
No tiene	14,81 ± 11,70		14,99 ± 11,55	
Tipo de cuidado				
Periódico	4,43 ± 4,11	0,001^b^	6,14 ± 4,69	0,005^b^
Continuo	13,03 ± 10,89		13,48 ± 10,27	
Enfermedad crónica				
Si	15,91 ± 11,75	< 0,001^b^	16,03 ± 10,77	< 0,001^b^
No	7,33 ± 6,78		8,51 ± 7,31	

^a^Test Kruskal Wallis

^b^Test Mann-Whitney U.

Siglas: BAI. Inventario de Ansiedad de Beck (Beck Anxiety Inventory); BDI. Inventario de Depresión de Beck (Beck Depression Inventory).

## DISCUSIÓN

En este estudio, analizamos exhaustivamente los niveles de ansiedad y depresión entre los cuidadores de pacientes con úlceras por presión que reciben servicios de atención domiciliaria, examinando sus asociaciones con diversas características demográficas y clínicas tanto de los pacientes como de los cuidadores. Los resultados contribuyen a la investigación existente al detallar el perfil de salud psicológica de los cuidadores de pacientes con úlceras por presión.

Las úlceras por presión en los pacientes que reciben atención domiciliaria se observaron con mayor frecuencia en la región sacra y fueron predominantemente de gravedad en estadio III. Este hallazgo concuerda con los datos que se suelen publicar en la literatura^(3,25)^. La mayor prevalencia de úlceras por presión en estadio avanzado puede estar relacionada con un tratamiento insuficiente de las úlceras por presión y una inmovilidad prolongada. Además, los pacientes que son monitoreados regularmente para el cuidado de las heridas en nuestro centro tienden a presentar úlceras en estadio más avanzado. Monitorear de forma más estrecha los factores de riesgo de úlceras por presión y la aplicación de estrategias de intervención temprana son fundamentales para prevenir la progresión a estadios más graves de las úlceras^(3)^. Los problemas nutricionales a los que se enfrentan estos pacientes pueden afectar negativamente al proceso de cicatrización de las heridas. Como resultado, el uso de métodos de alimentación alternativos, como la alimentación por sonda, en este grupo de pacientes puede estar asociado con una mayor necesidad tanto de tratamiento de las heridas como de cuidados generales^(26)^. En este estudio, aunque la mayoría de los pacientes se alimentaban por vía oral, el 23,8% se alimentaba mediante sonda PEG, lo que pone de relieve las necesidades sanitarias avanzadas de este grupo de pacientes. Los niveles más bajos de ansiedad observados entre los cuidadores de los pacientes alimentados mediante sondas NG y PEG sugieren una reducción del agotamiento emocional, posiblemente debido a la naturaleza sistemática y predecible de los cuidados que requieren estos pacientes.

En este estudio, los cuidadores de pacientes con úlceras por presión en estadio IV mostraron niveles más altos de ansiedad y depresión. Los síntomas depresivos entre los cuidadores aumentaron en correlación con la progresión de los estadios de las úlceras por presión. Estos hallazgos coinciden con el estudio de Fox^(27)^, que destaca la relación entre la gravedad de las úlceras por presión y el aumento del malestar psicológico, haciendo hincapié en el impacto de la gravedad de la enfermedad física en el bienestar mental. De manera similar, Qian et al.^(4)^ demostraron que el estrés de los cuidadores aumenta en relación directa con la progresión de las etapas de las úlceras por presión. Estos hallazgos indican que los cuidadores de pacientes con úlceras por presión en etapa avanzada experimentan niveles más altos de angustia psicológica, probablemente relacionados con los mayores desafíos asociados con las crecientes necesidades de atención de los pacientes. Por lo tanto, son esenciales las intervenciones de apoyo psicológico para los cuidadores, en particular los que atienden a pacientes con úlceras por presión en etapa avanzada.

La mayoría de los cuidadores de este estudio eran mujeres. Esta disparidad de género en las funciones de cuidado refleja la distribución de las responsabilidades de cuidado basada en el género y concuerda con los hallazgos de estudios anteriores^(28,29)^. En un estudio de Alhammadi et al.^(30)^, en el que los cuidadores de pacientes con úlceras por presión participaron en un programa educativo, el 91% de los cuidadores eran mujeres. Aunque las cuidadoras mostraron niveles más altos de síntomas de ansiedad y depresión en comparación con los cuidadores en este estudio, las diferencias no fueron estadísticamente significativas. Del mismo modo, los estudios publicados que no han encontrado diferencias significativas entre los sexos en el bienestar psicológico de los cuidadores coinciden con estos resultados^(29,31)^. A pesar de que las mujeres soportan una mayor carga emocional y física en el proceso de cuidado, las diferencias de género en los efectos psicológicos no siempre son evidentes. Esta observación podría atribuirse a las variaciones individuales en los mecanismos de afrontamiento o a las diferencias en la adaptación a las funciones de cuidado, influidas por factores personales y ambientales.

Se observó que los cuidadores que prestaban cuidados diarios durante muchas horas, ofrecían cuidados continuos, carecían de ingresos personales y padecían enfermedades crónicas presentaban niveles significativamente más altos de ansiedad y síntomas depresivos. Lee y Lee^(32)^ también han señalado una posible correlación entre el proceso prolongado y difícil de cuidar a personas con úlceras por presión y el aumento de los niveles de estrés y depresión entre los cuidadores. Qian et al.^(4)^ informaron de que los cuidadores de pacientes con úlceras por presión que padecían enfermedades crónicas y prestaban cuidados durante períodos más largos experimentaban un aumento de los niveles de fatiga y una disminución de su satisfacción con la vida. Estos hallazgos sugieren que la salud psicológica de los cuidadores se ve significativamente influida por factores como la duración de los cuidados, la situación económica y las enfermedades crónicas. Los cuidadores que experimentan períodos prolongados de cuidado, dificultades económicas y enfermedades crónicas parecen tener un mayor riesgo de sufrir un aumento del estrés, la fatiga y la depresión. Estos resultados subrayan la necesidad de programas de apoyo e intervenciones destinados a mitigar estos riesgos para los cuidadores que se enfrentan a situaciones de cuidado difíciles.

### Limitaciones

El diseño transversal de este estudio limita la capacidad de establecer relaciones causales, ya que los hallazgos reflejan solo un momento específico. Además, al tratarse de un estudio realizado en un solo centro, la generalización de los resultados es limitada y es posible que se hayan pasado por alto ciertos factores clínicos y psicosociales en el proceso de recolección de datos. El uso de escalas de autoinforme para evaluar la ansiedad y la depresión entre los cuidadores también introduce la posibilidad de sesgos.

## CONCLUSIÓN

En conclusión, los pacientes con úlceras por presión y sus cuidadores representan un grupo de alto riesgo que requiere una atención especializada por parte de los sistemas de salud. A medida que avanza la etapa de las úlceras por presión, aumenta la carga psicológica de los cuidadores. El impacto de la situación socioeconómica de los cuidadores y las enfermedades crónicas en sus niveles de ansiedad y depresión debe tenerse en cuenta a la hora de planificar intervenciones específicas para estas personas. Se recomienda encarecidamente ofrecer asesoramiento psicológico periódico e intervenciones preventivas a los cuidadores. Nuestra investigación pone de relieve la necesidad de estrategias más integrales para mejorar el tratamiento de las úlceras por presión y el apoyo a los cuidadores en el marco de los servicios de atención domiciliaria.

## Data Availability

Los conjuntos de datos generados y/o analizados durante el presente estudio están disponibles a través del autor correspondiente previa solicitud razonable.
